# Frequent Upregulation Of HER2 Protein In Hormone Receptor-Positive HER2-Negative Breast Cancer After Short-Term Neoadjuvant Endocrine Therapy

**DOI:** 10.21203/rs.3.rs-2777910/v1

**Published:** 2023-04-07

**Authors:** Lubna Naaz Chaudhary, Julie Jorns, Yunguang Sun, Yee Chung Cheng, Sailaja Kamaraju, John Burfeind, MaryBeth Gonyo, Amanda Kong, Caitlin Patten, Tina Yen, Chandler Cortina, Ebony Carson, Nedra Johnson, Carmen Bergom, Shirng-Wern Tsaih, Anjishnu Banerjee, Yu Wang, Inna Chervoneva, Elizabeth Weil, Christopher R Chitambar, Hallgeir Rui

**Affiliations:** Medical College of Wisconsin; Medical College of Wisconsin; Medical College of Wisconsin; Medical College of Wisconsin; MCW: Medical College of Wisconsin; MCW: Medical College of Wisconsin; MCW: Medical College of Wisconsin; MCW: Medical College of Wisconsin; MCW: Medical College of Wisconsin; MCW: Medical College of Wisconsin; MCW: Medical College of Wisconsin; MCW: Medical College of Wisconsin; MCW: Medical College of Wisconsin; MCW: Medical College of Wisconsin; MCW: Medical College of Wisconsin; MCW: Medical College of Wisconsin; MCW: Medical College of Wisconsin; Thomas Jefferson University; MCW: Medical College of Wisconsin; MCW: Medical College of Wisconsin; MCW: Medical College of Wisconsin

**Keywords:** Short-term neoadjuvant endocrine treatment, breast cancer, estrogen receptor, endocrine resistance

## Abstract

**Background.:**

Endocrine resistant metastatic disease develops in ~20–25% of hormone-receptor positive (HR+) breast cancer (BC) patients despite endocrine therapy (ET) use. Upregulation of HER family receptor tyrosine kinases (RTKs) represent escape mechanisms in response to ET in some HR+ tumors. Short-term neoadjuvant ET (NET) offers the opportunity to identify early endocrine escape mechanisms initiated in individual tumors.

**Methods.:**

This was a single arm, interventional phase II clinical trial evaluating 4 weeks (+/−1 week) of NET in patients with early-stage HR+/HER2-negative (HER2-) BC. The primary objective was to assess NET-induced changes in HER1–4 proteins by immunohistochemistry (IHC) score. Protein upregulation was defined as an increase of ≥1 in IHC score following NET.

**Results.:**

Thirty-seven patients with cT1-T3, cN0, HR+/HER2- BC were enrolled. In 35 patients with evaluable tumor HER protein after NET, HER2 was upregulated in 48.6% (17/35; p=0.025), with HER2- positive status (IHC 3+ or FISH-amplified) detected in three patients at surgery, who were recommended adjuvant trastuzumab-based therapy. Downregulation of HER3 and/or HER4 protein was detected in 54.2% of tumors, whereas HER1 protein remained low and unchanged in all cases. While no significant volumetric reduction was detected radiographically after short-term NET, significant reduction in tumor proliferation rates were observed. No significant associations were identified between any clinicopathologic covariates and changes in HER1–4 protein expression on multivariable analysis.

**Conclusion.:**

Short-term NET frequently and preferentially upregulates HER2 over other HER-family RTKs in early-stage HR+/HER2- BC and may be a promising strategy to identify tumors that utilize HER2 as an early endocrine escape pathway.

## Introduction

Adjuvant endocrine therapy (ET) provides significant recurrence-risk reduction as well as survival benefit to patients with hormone-receptor positive (HR+) breast cancer (BC). However, metastatic recurrences of endocrine resistant disease occur in about 20–25% of patients and remain a major cause of breast cancer mortality^1,2^. There is a continuum from early to late mechanisms of endocrine resistance in HR + BC, with some tumors exhibiting *de novo* resistance to endocrine treatments, while other tumors acquire resistance after an initial response^3,4^. Early escape and survival mechanisms of HR + BC in response to ET involve adaptive upregulation of growth factor receptor tyrosine kinases (RTKs), which promotes ligand-independent activation of estrogen receptors (ER) through MAP kinase signaling and phosphorylation of Ser11 of ER^5–7^. RTKs commonly upregulated in response to ET are members of the human epidermal growth factor receptor (HER) family, especially HER2 and epidermal growth factor receptor (EGFR)/HER1, but also HER3 and HER4, as well as FGFR1^4–8^. In a study of 240 metastatic HR+/HER2- BC patients on first-line ET, 25% converted to HER2-positive status at progression and had significantly shorter overall survival^9^. In another study, HER2 status switching, i.e. *ERBB2* gene amplification and/or overexpression, was observed in 12% (3/26) of HR+/HER2- tumors that became tamoxifen-resistant^10^. Intrinsic subtype switching with acquired HER2-positive status has also been reported in ~ 20% of metastatic recurrences of HER2- primary tumors^11^.

Neoadjuvant endocrine therapy (NET) can be used to downstage HR + tumors to potentially de-escalate surgery (increase rates of breast-conserving surgery; decrease rates of axillary node dissection in node-positive disease)^12–15^ in patients not felt to be ideal candidates for upfront chemotherapy. However, NET has more recently emerged as a strategy to assess changes in the tumor microenvironment and emerging resistance pathways as well as to determine tumor responses to additive targeted therapies in the neoadjuvant setting^16–18^. Most NET studies have included at least 3 months of treatment for tumor response assessments, however, changes in tumor proliferation have been reported as early as 2 weeks post-NET^19^.

Understanding and characterizing the underlying molecular features of endocrine resistance for more accurate response prediction and optimal clinical management to improve patient outcomes is of the utmost importance. Major clinical challenges to overcome late resistance include broad cancer cell heterogeneity and genetic diversity in advanced metastatic setting. Tailored targeting of early endocrine escape mechanisms used by individual HR + tumors, when metastatic burden is clinically undetectable and with lower genetic heterogeneity, will likely be more effective than later treatment directed at recurrent clinically overt metastases.

DESTINY Breast04^20^, a recent landmark study showed significant improvement in long term outcomes with HER2-directed therapy in patients with metastatic ‘HER2-low’ BC, which has traditionally been treated as HER2- BC. Given these findings, it is imperative to identify patients who may benefit from HER2- directed therapies. To identify targetable early endocrine escape mechanisms and potential candidates for HER2-directed therapies, we performed an interventional phase II clinical trial to assess molecular changes and tumor responses in early-stage HR+/HER2- BC patients treated with short-term NET, with the primary objective of measuring changes in HER1–4 protein levels with NET.

## Methods

### Study Design and Patients.

A phase II single arm, interventional clinical trial was completed to evaluate changes in molecular biology and tumor responses in patients treated with short-term NET. The primary objective of the study was to assess NET-induced changes in HER1–4 protein expression levels in tumors, and their association with post-NET cancer cell proliferation rate dichotomized Ki-67 positivity as low (< 10%) or high (≥ 10%). Secondary objectives included assessment of radiographic responses and other molecular markers. We enrolled women aged 18 years or older with HR+/HER2- BC with histologically confirmed operable and clinically or radiographically measurable lesions (cT1-T3, cN0, cM0). Estrogen and/or progesterone receptor positivity was defined as ≥ 1% nuclear staining by immunohistochemistry (IHC) and HER2/neu-negative by IHC or fluorescence *in situ* hybridization (FISH) according to the current ASCO/CAP guidelines^21,22^. Patients had to have an ECOG performance status of ≤ 2. Patients with bilateral BC and those with multifocal or multicentric disease were eligible. Patients were excluded if they had lymph node-positive or distant metastatic BC, purely noninvasive BC (i.e., ductal carcinoma *in situ*, lobular carcinoma *in situ*), HER2-positive BC, history of any malignancy except non-melanomatous skin cancer or carcinoma *in situ* of the cervix within 2 years, or were pregnant or actively breast feeding. All patients provided written informed consent. This study was performed in line with the principles of the Declaration of Helsinki. The Institutional Review Board and the Protocol Review and Monitoring Committee of the Medical College of Wisconsin approved the study.

### Procedures.

**Figure 1:**
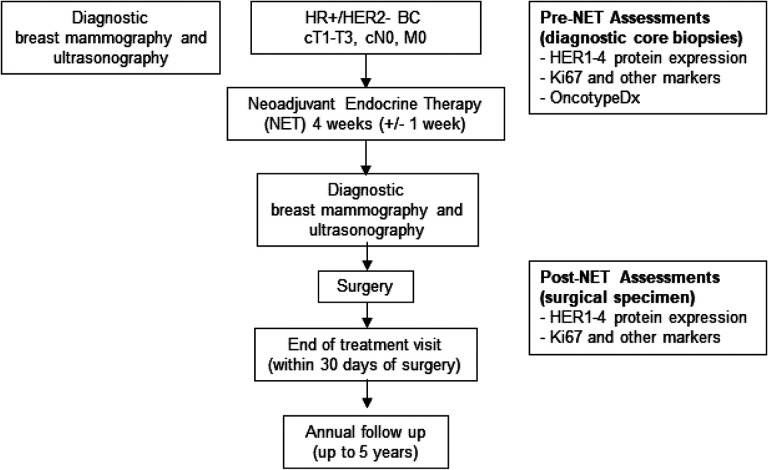
Study Schema

Patients were treated with NET for 4 weeks (+/− 1 week) prior to surgery, with dosing continuing until the day of surgery (+/− 2 days). Choice of NET regimen [tamoxifen or aromatase inhibitors (AIs)], based on menopausal status, medical conditions and patient preference, was at the discretion of the treating physicians. Standard daily dosing was used without any dose reductions or modifications (tamoxifen 20mg, anastrozole 1mg, letrozole 2.5mg, exemestane 25mg). Ovarian suppression with gonadotropin-releasing hormone (GnRH) analogues was allowed for premenopausal women. Cytochrome P450 2D6 (CYP2D6) inhibitors were prohibited with tamoxifen use. Initial diagnostic core biopsies were used for pre-treatment biomarker assessments and surgical tumor specimens were used for post-treatment assessments. Study Schema is shown in Fig. 1. Patients were followed for 30 days after surgery to record any adverse events from treatment. Adverse events were assessed according to Common Terminology Criteria for Adverse Events version 5.0. After tumor resection, patients were treated with radiation and/or chemotherapy according to standard-of-care treatment guidelines. Pre-NET biopsy specimens were used for OncotypeDx testing to aid in adjuvant chemotherapy decision making when indicated. ET will continue in the adjuvant setting for a period of at least 5 years. Patients will be followed for at least five years from completion of study to assess patient outcomes, i.e., ipsilateral, contralateral or distant recurrences.

### Clinical Assessments.

Patients underwent diagnostic breast mammography and ultrasonography at the time of diagnosis and again within 5 days prior to surgery. For clinical radiographic tumor measurements, the largest bi-dimensional measurements and when possible, three-dimensional measurements were recorded. Radiographic tumor responses were assessed using World Health Organization Response Evaluation Criteria in Solid Tumors (WHO): Complete response (disappearance of tumor), Partial response (≥ 50% decrease in the product of the bi-dimensional measurements of the tumor), No change (50% decrease in total tumor size cannot be established nor has a 25% increase in the size of the lesion been demonstrated), or Progressive disease (≥ 25% or greater increase in the total tumor size).

### Pathology Assessments.

Among the 37 patients, one patient had pathological complete response (pCR) and a second patient had too few residual cancer cells post-NET to measure HER proteins. Analyses of paired molecular tumor data on pre- and post-NET specimens were therefore limited to 35 patients. Standardized immunohistochemistry (IHC) protocols for estrogen receptor (ER) (6F11, Leica Biosystems, Buffalo Grove, IL), progesterone receptor (PR) (16, Leica Biosystems, Buffalo Grove, IL), HER1 (EGFR H11, Agilent, Santa Clara, CA), HER2 (4B5, Ventana, Roche Diagnostics, Indianapolis, IN), HER3 (HER3, Cell signaling, Danvers, MA) and HER4 (erbB4/HER4, MilliporeSigma, Burlington, MA) as well as HER2 dual probe FISH (PathVysion, Abbott, Abbott Park, IL) testing if HER2 IHC score was equivocal (2+), were performed in a College of American Pathologists (CAP)-accredited laboratory and all stains were visually evaluated by a board certified breast pathologist (JMJ) to assess biomarker status in the pre- and post-treatment tumor specimens. IHC for HER1 (EGFR), HER3 and HER4 was interpreted visually, with HER1 IHC interpreted as positive if there was membranous staining and HER3 and HER4 interpreted as positive if there was cytoplasmic and/or membranous staining, with 0 being negative (< 1%) and 1+, 2 + or 3 + scores matching the intensity and cutoffs (10% tumor staining) used by the current HER2 guidelines^22^. HER1–4 protein upregulation was defined as an increase of ≥ 1 in IHC score (ordinal 0,1,2,3), whereas downregulation conversely was defined as a decrease of ≥ 1 in IHC score.

Histopathological responses were assessed by change in tumor proliferation and cellularity. Tumor proliferation protein Ki-67 (MIB-1, Agilent, Santa Clara, CA) on pre- and post-treatment specimens was assessed by quantitative IHC. For Ki-67 quantification image analysis, QuPath (version 0.3.0, build 2021) software (Queen’s University Belfast, Belfast, Northern Ireland; https://qupath.github.io) was used for the cell detection, segmentation, objective classification (cancer and stroma) and determination of the percent of Ki-67-positive cancer cells. Tumor cellularity and tumor infiltrating lymphocytes (TILs) in pre- and post-treatment tumor specimens were assessed visually by pathologist according to Residual Cancer Burden guidelines^23^ and the International TILs Working Group guidelines^24^, respectively

### Statistical analyses.

We planned a sample size of 37 patients to achieve at least 80% power at significance level of 0.05, when testing the one-sided one sample hypothesis that the proportion of tumors with NET-associated upregulation of one or more HER proteins (HER1–4) is at least 50% versus the null hypothesis that the proportion is no larger than 30%. One patient had two ipsilateral HR + tumors with distinct histologies. Since two tumors in one patient cannot be regarded as independent, biostatistician blinded to marker data determined that the tumor with highest post vs. pre-NET HER1–4 protein change should be included in the analyses, with larger tumor size serving as a second selection criterion in case of a tie. Chi-square, Wilcoxon rank-sum tests and t-tests were performed where appropriate. Linear regression multivariable analysis was performed to evaluate the association of covariates (age, BMI, menstrual status, tumor grade, histology, pre- and post-NET changes in tumor size, Ki67, ER%, PR%, cellularity and TILs) with the primary outcome of changes in HER1–4 protein levels. All analyses were performed using SAS version 9.4 (Cary, NC).

## Results

### Patient characteristics.

Between March 2018 and February 2020, 37 women diagnosed with localized invasive HR+/HER2- BC were enrolled. Patient and pretreatment tumor characteristics are summarized in Table 1. Most patients were post-menopausal (83.8%). Median age at diagnosis was 64 years (range 42–81) and median BMI was 28.3 kg/m2 (19.3–55.1). Overall, the majority of tumors were grade 1 (42.1%) or grade 2 (47.4%) and had ductal histology (70%). Median tumor size clinically at diagnosis was 1.3 cm (0.5–7.7). Median pathological tumor size at surgery was 1.2 cm (0.0–4.0). One patient had a complete pathologic response (pCR) at surgery with no residual tumor tissue available while another patient had pT1mi disease resulting in material insufficient for repeat HER1–4 testing, resulting in paired pre- and post-NET tumor HER1–4 tumor assessments for 35 patients. One patient had two ipsilateral HR + tumors with distinct histology (invasive ductal carcinoma and invasive lobular carcinoma; see Statistical Methods section). Seven patients had pN1 disease at surgery (six with 1 lymph node involved, one with pN1mi). OncotypeDx^®^ Recurrence Score (RS) was performed on diagnostic core biopsies in 27 patients with low RS (≤ 15) in 10 patients, intermediate RS (16–25) in 8 patients and high RS (≥ 26) in 9 patients.

### NET-associated upregulation of HER1–4 protein expression.

**Figure 2:**
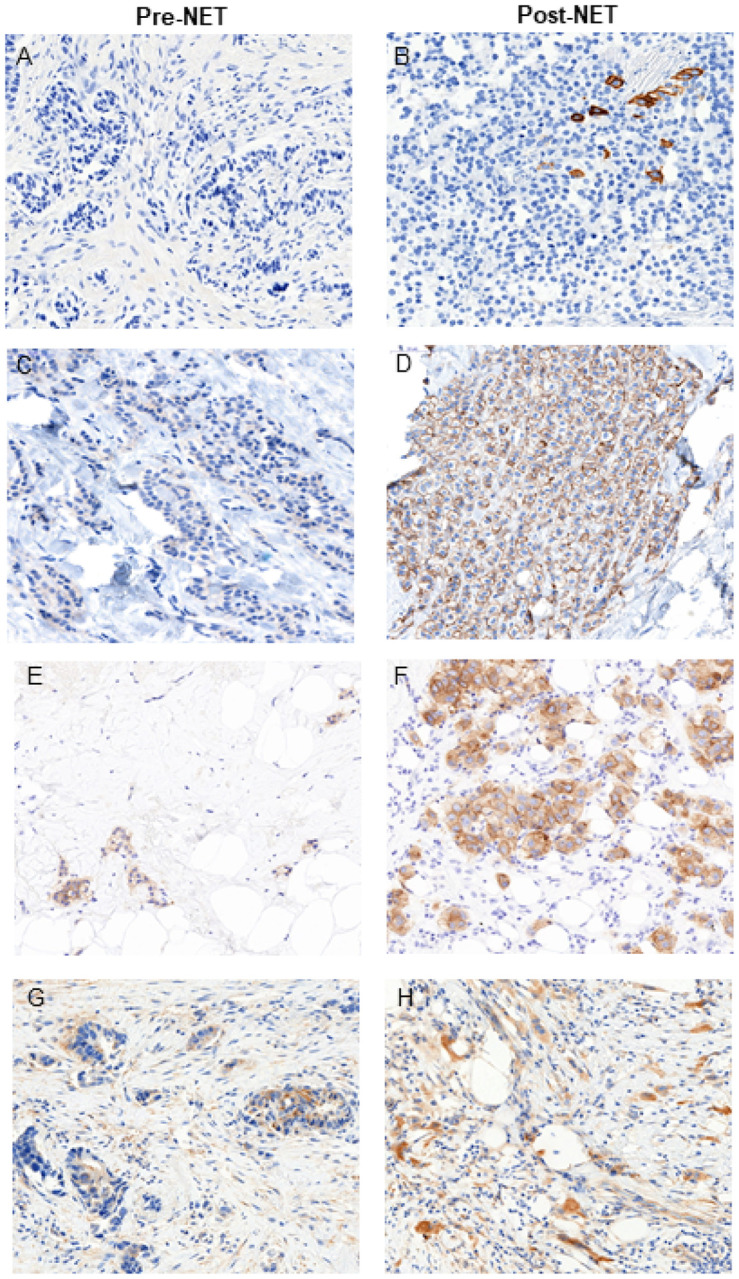
Immunohistochemistry of HER family of proteins in early-stage HR+/HER2- breast cancer before and after short-term neoadjuvant endocrine therapy. (A, B) - HER1/EGFR, (C, D) - HER2, (E, F) - HER3, and (G, H)-HER4. Note: although minor emergence of HER1/EGFR expression was observed, in no case was HER1- positive cells >1%.

**Figure 3:**
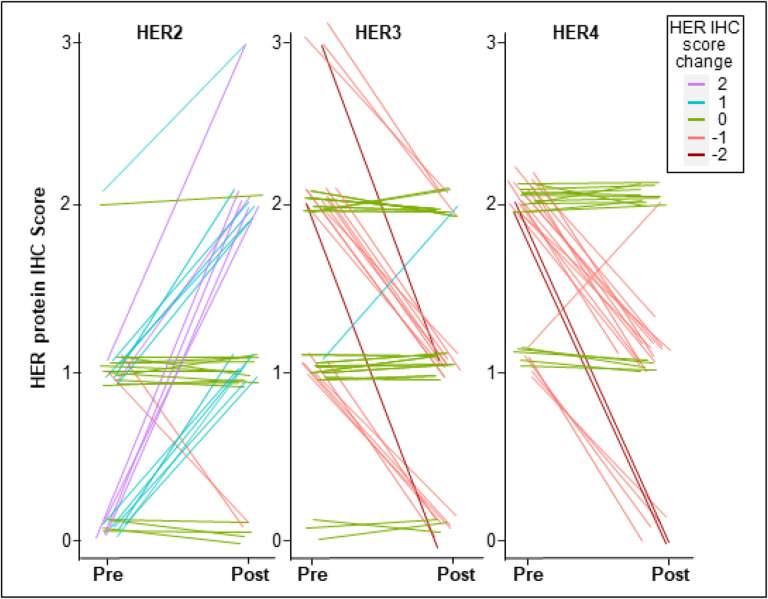
Pre- and Post-NET changes in HER2, HER3 and HER4 in early-stage HR+/HER2- breast cancer.

IHC scoring was used to identify patients with tumors displaying upregulation of HER1–4 protein levels in cancer cells from pre-NET biopsies to post-NET surgical resections. Collectively, upregulation of one or more of the HER proteins in tumors after ~ 4 weeks of NET were detected in 17 of the 35 patients with evaluable tumors (48.6%; p = 0.025; exact binomial 95% CI of 31.4%−66.0%; Fig. 2, Fig. 3**, Supplementary Table 1**). Intriguingly, HER2 was upregulated in all 17 cases that had upregulation of any HER protein, with dual upregulation of HER3 in one case, dual upregulation of HER4 in another case, while HER1 protein levels were low and remained unchanged in all 35 cases. In contrast, HER2 protein downregulation was observed in only two cases (2/35; 5.7%), while HER3 and HER4 proteins were each downregulated in 19 cases (54.2%). Bivariate Spearman rank correlation revealed no significant associations between changes in HER1–4 protein scores after short-term NET. In addition to upregulation of HER2 protein in 17 cases, one additional tumor with equivocal HER2 IHC score of 2 + both pre- and post-NET showed *ERBB2* gene amplification post-NET. In total, three of the 35 patients (8.6%) with evaluable HER2 scores pre- and post-NET converted from HER2-negative to HER2-positive status (IHC 3 + or FISH amplified) at surgery and were recommended adjuvant trastuzumab-based treatment. Of the 14 cases with HER2 IHC score of 0 at baseline, post-NET 10 cases upregulated to IHC 1 + or 2+, qualifying as “HER2-low”. No significant associations were identified between any clinicopathologic covariates and changes in HER1–4 protein expression on multivariable analysis.

### NET-associated changes in Ki-67 and PR.

**Figure 4:**
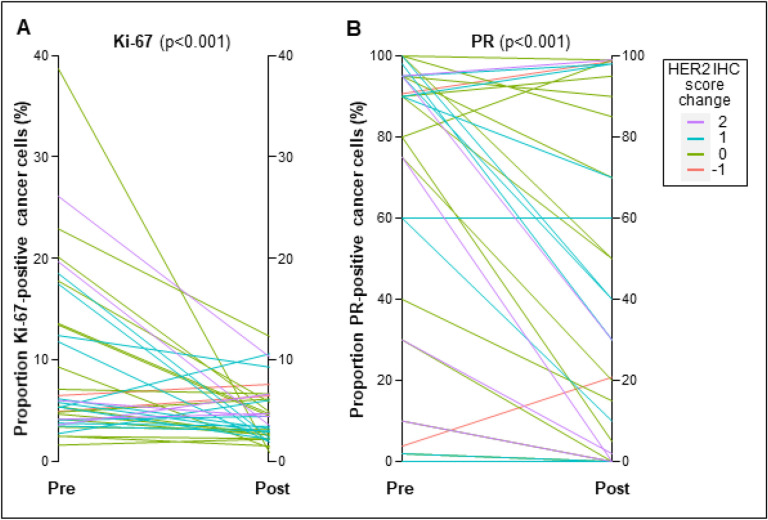
NET-induced reductions in Ki-67 and PR in early-stage HR+/HER2- breast cancer.

Significant reductions in cancer cell proliferation rates were induced by short-term NET, with median Ki-67-positivity of 9.7% before treatment and 4.5% after treatment (p < 0.001; Fig. 4A). NET led to a decrease in tumor Ki-67-positivity in 74.3% of cases [median decrease of 3.14 percentage points (range 0.22–37.8)] and an increase in 25.7% of tumors [median increase of 1.29 percentage points (range 0.37–5.32)]. High cancer cell Ki-67-positivity ≥ 10% was only detected in 3 cases (8.6%) post-NET and was not associated with changes in HER protein levels. Intermediate tumor Ki-67-positivity between 2.7–10% was observed in 21 cases (60.0%) post-NET, whereas Ki-67-positivity was ≤ 2.7% in 11 cases (31.4%), consistent with complete cell cycle arrest^19^. While ER-positivity remained unchanged between pre- and post-treatment specimens, mean PR-positivity was reduced from 62.1–42.1% after short-term NET (p < 0.001, Fig. 4B), consistent with functional ER pathway disruption.

### Radiographic responses.

Median tumor size change was − 7.6% (range − 60.3 to 44.3) by ultrasonography and − 5.8% (range − 49 to 59) by mammography with short-term NET, however, these changes were not statistically nor clinically significant (p = 0.83 and p = 0.85, respectively). Pre-treatment low tumor grade and PR-positivity were significantly associated with tumor volume reduction radiographically (p = 0.006 and p = 0.02 respectively), while higher OncotypeDx RS was a negative predictor of radiographic response (p = 0.02). No significant correlation of radiographic response was detected with changes in HER1–4 scores. When assessed by WHO criteria, most patients had no change on radiographic response assessment, one had a partial response and 3 patients had disease progression.

### Adverse Events.

NET was associated with a favorable toxicity profile. No adverse events (AEs) ≥ grade 3 were seen in this study. Incidence of grade 1 and 2 AEs were low (2.7% and 5.5%, respectively) and included arthralgias, insomnia, fatigue, hot flashes, nausea, constipation, and headaches. No discontinuation due to AEs or complications were seen.

## Discussion

In this study, we treated 37 women with early-stage HR+/HER2- BC with short-term NET. Paired specimens for pre- and post-NET tumor HER1–4 assessments were available for 35 tumors and we identified upregulation of HER2 protein in 17 tumors (48.5%), including two that converted to HER2 IHC3 + status at surgery. In addition, one additional tumor with equivocal HER2 IHC score of 2 + both pre- and post-NET showed *ERBB2* gene amplification post-NET. The *ERBB2* gene amplification uncovered after short-term NET in this tumor is assumed to represent tumor heterogeneity with pre-existing HER2 gene amplification that was likely not captured in the smaller biopsy sample. Regardless, HER2 analyses after short-term NET identified a total of three patients (8.6%) that were recommended adjuvant trastuzumab-based chemotherapy. In contrast to the observed frequent upregulation of HER2 protein, short-term NET was associated with frequent downregulation of protein levels of HER3 and/or HER4 in more than half of the cases, while upregulation of either HER3 or HER4 protein was rare. HER1/EGFR protein levels were low and generally remained unchanged in these early-stage HR + tumors.

Significant and selective upregulation of HER2 protein, in response to short-term NET is suggestive of compensatory activation of HER2 signaling in nearly half of the HR+/HER2- tumors. This finding is important because signaling via HER2 alone or with its dimerization partners HER1 or HER3 represents a well-established endocrine resistance mechanism based on experimental studies of HR + breast cancer cell lines^25^, *in vivo* tumor studies^26^, and most importantly by frequent HER2-upregulation in recurrent metastases after anti-estrogen therapy^10,27–30^. Likewise, HR+/HER2-positive tumors have poor prognosis compared to HR+/HER2- tumors and frequently display inherent or rapidly acquired endocrine resistance^31,32^. While HR+/HER2-positive tumors clinically respond at least as well to NET as HR+/HER2- tumors in terms of volumetric reduction^33,34^, data from two clinical trials of standard NET of 4–6 month duration showed that cancer cell proliferation rates (Ki-67-positivity) after NET remained significantly higher in ER+/HER2-positive tumors^35^.

The tumor proliferation marker Ki-67 is a useful and reliable measure of early response or resistance to treatment and is being used in NET trials to guide developmental therapeutics. Reduction in Ki-67 as early as 2 weeks post-NET has been reported in endocrine sensitive tumors while endocrine resistant tumors had a less impressive reduction highlighting the sensitivity of Ki-67 measurements in NET response assessments^19,36,37^. The IMPACT trial^38^ showed a statistically significant 85% reduction of mean cancer cell Ki-67 positivity rate in the HER2-negative group compared to a 45% reduction in the HER2-positive group despite good clinical responses seen in the HER2-positive group, which suggests rapid emergence of HER2-mediated resistance. While short-term NET was associated with significant reductions in Ki-67 in our study, no statistically significant associations between post-NET Ki-67 and levels of HER2 or other HER family protein levels were seen. Based on the observations of frequent upregulation of HER2 in HR+/HER2- BC, short-term NET while patients are awaiting surgery may therefore identify HR+/HER2- tumors that engage HER2-signaling to overcome ET.

In this study, downregulation in both HER3 and/or HER4 was seen in 54.2% of tumors in response to short-term NET. HER3 overexpression has been associated with HER2-mediated tamoxifen resistance in preclinical and clinical studies and downregulation of HER3 has been shown to inhibit HER2 associated proliferation and tumorigenesis^29^. Nuclear HER4 intracellular domain (ICD) functions as a potent ER co-activator and promotes the proliferation of ER + breast tumor cells^39^ whereas cytosolic HER4 ICD has antiproliferative and pro-apoptotic activity including tamoxifen induced apoptosis^40^. Pre-clinical studies have shown that the presence of HER4 in HER2-positive BC cells results in reduced proliferation and increased apoptosis, possibly indicating that HER4 antagonizes HER2 signaling activity^41,42^. Consequently, loss of HER4 may be associated with tamoxifen resistance in patients with BC. The significance of HER3 and HER4 downregulation will require further study.

Limitations of our study include small sample size and the possibility of tumor heterogeneity, clonality, and tissue biopsy sampling impacting the histological results. It is conceivable that in select cases there was pre-existing focal HER2-positive disease that was not detected by biopsy sampling. However, upregulation of HER2 protein in almost half of the cohort is unlikely to be secondary to tumor heterogeneity or tissue biopsy sampling differences.

In conclusion, this pilot study demonstrates the possibility of identifying early endocrine resistance mechanisms by using a cost-effective, well-tolerated and practical strategy of short-term NET. This finding creates an opportunity for in-vivo analysis of the tumor microenvironment and exploration of biomarker development to provide personalized care by tailoring adjuvant treatments and improving patient outcomes.

## Figures and Tables

**Table 1:** Patient and tumor characteristics

Patient characteristics	N = 37
Median Age (range)	64 yrs (42–81)
Median BMI (range)	28.3 (19.3–55.1)
Post-menopausal	31 (83.8%)
**Neoadjuvant Endocrine Therapy**	
Aromatase inhibitor**	28 (75.6%)
Tamoxifen	9 (24.4%)
**Surgery**	
Lumpectomy	26 (70%)
Mastectomy	11 (30%)
**Adjuvant Therapy**	
Radiation	24 (65%)
Chemotherapy	10 (27%)
No radiation, no chemotherapy	3 (8%)
**Node status at surgery**	
pN0	30 (81%)
pN1	7 (19%)
Tumor characteristics	N = 38*
Median clinical tumor size (range)	1.3cm (0.5–7.7)
Median tumor size at surgery (range)	1.2cm (0.09–4)
**Histology**	
Invasive ductal	26 (68%)
Invasive lobular	12 (32%)
**ER/PR status**	
ER+/PR+	32 (84%)
ER+/PR−	6 (16%)
**Tumor grade**	
1	16 (43.2%)
2	18 (47.4%)
3	4 (10.5%)

* One patient multifocal disease (two HR + foci with distinct histology)

** One patient treated with aromatase inhibitor and GnRH analog

Acknowledgement and Funding information:

## Data Availability

The datasets generated during and/or analyzed during the current study are available from the corresponding author on reasonable request.
